# *Lactobacillus reuteri* Alleviates Hyperoxia-Induced BPD by Activating IL-22/STAT3 Signaling Pathway in Neonatal Mice

**DOI:** 10.1155/mi/4965271

**Published:** 2024-12-09

**Authors:** Meiyu Zhang, Decai Li, Liujuan Sun, Yu He, Qingqing Liu, Yi He, Fang Li

**Affiliations:** ^1^National Clinical Research Center for Child Health and Disorders, Ministry of Education Key Laboratory of Child Development and Disorders, Chongqing Key Laboratory of Child Rare Diseases in Infection and Immunity, Department of Neonatology Children's Hospital of Chongqing Medical University, Chongqing 400015, China; ^2^Department of Pediatrics Chongqing Health Center for Women and Children, Department of Pediatrics Women and Children's Hospital of Chongqing Medical University, Chongqing 401147, China

**Keywords:** bronchopulmonary dysplasia, IL-22/STAT3 signaling pathway, *L. reuteri*, lung microbiota

## Abstract

Bronchopulmonary dysplasia (BPD) is the most common chronic respiratory disease in preterm infants. Little is known about the regulatory effect of lung *Lactobacillus* and its mechanism in BPD. This study explored the effect of *L. reuteri* on hyperoxia-induced mice lung injuries and examined whether *L. reuteri* played a role via the IL-22/STAT3 pathway. We found that the intranasal administration of *L. reuteri* and its tryptophan metabolite indole-3-aldehyde (3-IAld) ameliorated hyperoxia-induced mice lung BPD-like changes, deceased proinflammatory cytokines (IL-1*β*, IL-6, and TNF-*α*), and increased the levels of surfactant-associated protein C (SPC), aquaporin 5 (AQP5), and vascular endothelial growth factor receptor 2 (VEGFR2, also known as FLK-1). Furthermore, *L. reuteri* and 3-IAld increased the expression of IL-22. IL-22 was also confirmed to ameliorate hyperoxia-induced mice lung pathological changes, and the protective effects of *L. reuteri* could be inhibited by anti-IL-22 neutralizing antibody. Finally, we confirmed STAT3 activation by IL-22 in MLE-12 cells. In summary, our study confirmed *L. reuteri* alleviated hyperoxia-induced lung BPD-like changes in mice by activating the IL-22/STAT3 signaling pathway via IL-22 production. Probiotics *Lactobacillus* is a potential treatment for hyperoxia-induced lung injury in newborns.

## 1. Introduction

Bronchopulmonary dysplasia (BPD) is the most common chronic respiratory disease in preterm infants; this disease requires respiratory support and leads to significant lifelong consequences [1]. The predominant pathological changes observed in BPD include alveolar simplification and dysregulated vascular growth [2]. Although neonatal management has significantly improved over the past few decades, the morbidity and mortality of BPD in very preterm newborns are still increasing [3]. BPD occurs secondary to genetic–environmental interactions in the immature lung, and multiple factors contribute to BPD [4–6]. Although the specific etiology of BPD remains unclear, evidence has shown that dysregulation of the lung microbiome might play an important role in BPD development in preterm infants [7, 8] and that modifying the airway microbiome might reduce the risk of BPD [9]. In particular, preterm infants who later develop BPD have a decreased abundance of *Lactobacillus* in the early airway microbiome [10].

Probiotic *Lactobacillus* strains have been found to be effective at treating acute and chronic respiratory diseases, such as respiratory distress syndrome [11], acute lung injury [12] obstructive pulmonary disease [13], asthma [14], and cystic fibrosis [15]. In addition, there are reports that *lactobacillus* can modulate lung immunity and protect against infections caused by the influenza virus [16], *Pseudomonas aeruginosa* [17] and *Haemophilus influenzae* [18]. In germ-free mice, lung Lactobacillus transplantation was proven to improve alveolar development [19].

Although *Lactobacillus* has been widely used in the prevention and treatment of respiratory diseases in animal experiments, little is known about the effect and mechanism of *Lactobacillus* on BPD, and there are no reports about the application of *L. reuteri* in the prevention and treatment of BPD. Indole-3-aldehyde (3-IAld) is a *Lactobacillus* tryptophan metabolite that can activate aryl hydrocarbon receptors (AhRs) in the intestine, promoting the secretion of IL-22, and the growth of epithelial cells in the intestine [20].

This study aims to investigate whether *L. reuteri* plays a protective effect on BPD and its possible mechanism. We hypothesized that *L. reuteri* could ameliorate BPD-like injuries and promote the development of alveolar and pulmonary vessels by stimulating the secretion of IL-22.

## 2. Materials and Methods

### 2.1. Animal Experiments

C57BL/6 mice (6–8 weeks old, specific-pathogen-free [SPF]) were purchased from the Animal Research Centre of Chongqing Medical University. All mice were provided sterile mouse chow and water ad libitum and were maintained on a constant 12/12-h light/dark cycle. Two female mice and one male mouse were kept in a cage to produce newborn mice, and the female mice were allowed to natural delivery after mating. All procedures were reviewed and approved, per IACUC guidelines, by the Ethics Committee of Children's Hospital of Chongqing Medical University (IACUC Issue No: CHCMU-IACUC202211227001).

The newborn mice were randomly assigned to normoxia (21% O_2_) or hyperoxia (85% O_2_) treatment groups from postnatal day P3–P17. The nursing mothers were rotated between the hyperoxia treatment and normoxia treatment every 24 h to prevent oxygen toxicity.

Newborn mice under normoxic conditions were randomly grouped into control, control+L and control+3-IAld groups, and newborn mice under hyperoxic conditions (85% O_2_) were randomly grouped into hyperoxia, hyperoxia+L, hyperoxia+3-IAld, hyperoxia+IL-22, and hyperoxia+L+anti-IL-22 groups, with 5–7 mice per group. The mice in the control/hyperoxia+L groups were intranasally instilled with *L. reuteri* (10^8^ CFU/per mouse, strain BNCC254476, China) [21]. *L. reuteri* was grown in MRS agar media at 37°C. The mice in the control/hyperoxia+3-IAld groups were intranasally instilled with 3-IAld (18 mg/kg; Abmole, China) [22]. The mice in the control and hyperoxia groups were intranasally instilled with PBS. However, murine recombinant IL-22 (1 mg/kg; Prime Gene, China) [23] and an anti-IL-22 neutralizing antibody (8 mg/kg; Invitrogen, USA) [24] were administered by intraperitoneal injection. The animals in each group were treated every 48 h from P3–P17. The neonatal mice were euthanized on postnatal day 17, and lung tissues were obtained for further analysis.

### 2.2. Cell Culture

MLE-12 cells (iCell, China) were cultured at 37°C in complete MLE-12 medium (Pricella, China) supplemented with 2% fetal bovine serum, 10 nM hydrocortisone, 10 nM *β*-estradiol, and 1% penicillin and streptomycin in 5% CO_2_. To establish a hyperoxia-induced lung injury model in vitro, cells (1 × 10^6^ cells per well in a six-well plate) were cultured under hyperoxic conditions (95% O_2_/5% CO_2_) for 24 h. After 24 h of treatment with IL-22 (50 ng/mL), anti-IL-22(1 μg/mL), and PBS, protein was obtained for further analysis.

### 2.3. Histological Tests (Hematoxylin–Eosin Staining)

After fixation with 4% paraformaldehyde and embedding in paraffin, lung tissues were cut into 4 µm pathological sections for staining. Hematoxylin and eosin were used to stain the tissue sections for further pathological analysis. Pathological changes in each group were assessed by the mean linear intercept (MLI) and the radical alveolar counts (RACs). MLI assesses average alveolar size using the following formula: MLI = total length/alveolar septal number, 10 lines were used in each region to measure the MLI, RAC is used to assess the number of alveolar. Both MLI and RAC take five fields from each slice to calculate the mean.

### 2.4. Enzyme-Linked Immunosorbent Assay (ELISA)

The concentrations of IL-1*β*, IL-6, TNF-*α*, and IL-22 in mouse lung tissue homogenates were quantified by specific ELISA kits (IL-1*β* and IL-22; 4A BIOTECH, China; IL-6 and TNF-*α*; Signalway Antibody, China) following the manufacturer's instructions. Optical densities were determined by utilizing a microtiter plate reader (Thermo Fisher Scientific, USA).

### 2.5. Western Blot Analysis

Western blot was used to assess the relative levels of surfactant-associated protein C (SPC), aquaporin 5 (AQP5), and FLK-1 in mouse lung tissues and of STAT3 and pSTAT3 in MLE-12 cells. Total protein was extracted from lung tissues and cells by utilizing RIPA lysis buffer (Beyotime, China) supplemented with protease and phosphatase inhibitors (Solarbio, China). The concentration of total protein was measured by a bicinchoninic acid (BCA) assay (Beyotime, China). After electrophoresis, protein separation, protein transfer, and membrane blocking, the membrane was incubated at 4°C overnight with the following primary antibodies: anti-SPC (1 : 1000; Proteintech, China), anti-AQP5 (1 : 1000; Proteintech, China), anti-FLK-1 (1 : 1000; Cell Signaling Technology, USA), anti-STAT3 (1 : 500; Cell Signaling Technology, USA), and anti-pSTAT3 (1 : 500; Cell Signaling Technology, USA). Subsequently, the membranes were incubated with HRP-conjugated goat anti-rabbit IgG (1 : 5000, ZSBio, China) after three washes. *β*-Actin (1 : 10,000; ZENBio, China) was used as an internal control. An Odyssey infrared imaging system (Bio-Rad, USA) and ImageJ software were used to obtain and analyze the images.

### 2.6. Quantitative Reverse Transcription-PCR (RT-qPCR)

Total mRNA was extracted from lung tissues by using an RNA extraction kit (SteadyPure Mag mRNA Kit, Accurate Biology, China) and reverse transcribed into cDNA by utilizing a RT kit (Evo M-MLV Mix Kit with DNA Clean for qPCR, Accurate Biology, China). A StepOnePlus real-time PCR System (ABI, USA) was used to quantify the relative expression levels of SPC, AQP5, and VEGF in lung tissues, and GAPDH mRNA expression was used as an internal control. Each sample was analyzed in triplicate, and the results represent three independent experiments. The sequences of primers used in the study are listed in Table 1.

### 2.7. Cell Counting Kit-8 (CCK8) Proliferation Assay

A CCK-8 proliferation assay (Solarbio, China) was used to measure cell viability, and three replicates were performed. Cells were seeded in 96-well plates at a density of 5 × 10^3^ cells/well. After 24 h of culture, 24 h of hyperoxia, and 24 h of IL-22 stimulation, CCK-8 solution (10 μL/well) was added to each well. The optical density (OD) of the CCK-8 reagent was detected by a microplate reader (Thermo Fisher Scientific, USA) after 0.5 h of incubation at 37°C.

### 2.8. Statistical Analysis

In this study, all the statistical analyses were performed using GraphPad Prism, version 9.5, and all the data were statistically compared. The measurement data are expressed as the mean ± standard deviation (x ± s), and *p* < 0.05 was considered to indicate statistical significance.

## 3. Results

### 3.1. *L. reuteri* and 3-IAld Ameliorate Lung Inflammation and BPD-Like Changes in Hyperoxia-Induced Mice

**Figure 1:**
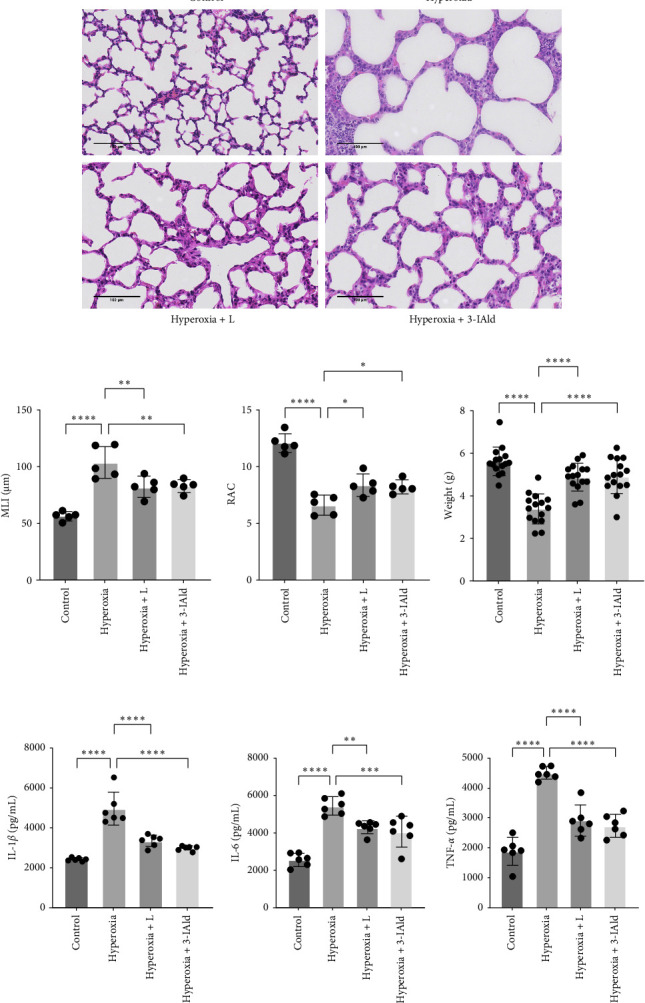
*L. reuteri* ameliorates hyperoxia-induced BPD-like changes and lung inflammation in mice. (A) H&E staining of lung tissue from the control, hyperoxia, hyperoxia+L, and hyperoxia+3-IAld groups (400×). (B) MLI and RAC analyses were performed to evaluate alveolarization in each group. The MLI and RAC values represent the average of five visual fields in the same section (*n* = 5). (C) Comparison of body weight was performed on P17 (*n* = 15). (D) ELISAs of IL-1*β*, IL-6, and TNF-*α* levels in each group (*n* = 6). The data are presented as means ± SDs. All the statistical analyses were performed by ordinary one-way ANOVA following normality and homogeneity tests. *⁣*^*∗*^*p* < 0.05, *⁣*^*∗∗*^*p* < 0.01, *⁣*^*∗∗∗*^*p* < 0.001, *⁣*^*∗∗∗∗*^*p* ≤ 0.0001. BPD, bronchopulmonary dysplasia; ELISAs, enzyme-linked immunosorbent assays; MLI, mean linear intercept; RAC, radical alveolar count.

To explore the effect of lung *lactobacillus* on hyperoxia-induced lung injury, *L. reuteri* and 3-IAld were intranasally administrated to newborn C57BL/6 mice. As shown by H&E staining, compared with the control group, mice in the hyperoxia group exhibited significant alveolar simplification, also called BPD-like changes (Figure 1A). The MLI and RAC revealed enlarged alveolar size and a reduced alveolar number in the hyperoxia group (Figure 1B). Compared with the hyperoxia group, lung pathological changes of mice in the hyperoxia+L group and hyperoxia+3-IAld group were alleviated, with a lower MLI and greater RAC. In addition, compared with the hyperoxia group, the weight of mice in the hyperoxia+L group and hyperoxia+3-IAld group increased significantly (Figure 1C). The ELISA results revealed increased protein expression of the proinflammatory cytokines IL-1*β*, IL-6, and TNF-*α* in the hyperoxia group than those in control group (Figure 1D). Compared with the hyperoxia group, the levels of proinflammatory cytokines IL-1*β*, IL-6, and TNF-*α* in the hyperoxia+L group and hyperoxia+3-IAld group significantly decreased.

### 3.2. *L. reuteri* and 3-IAld Increase the Expression Levels of SPC, AQP5, and FLK-1 in Hyperoxia-Induced Mice

**Figure 2:**
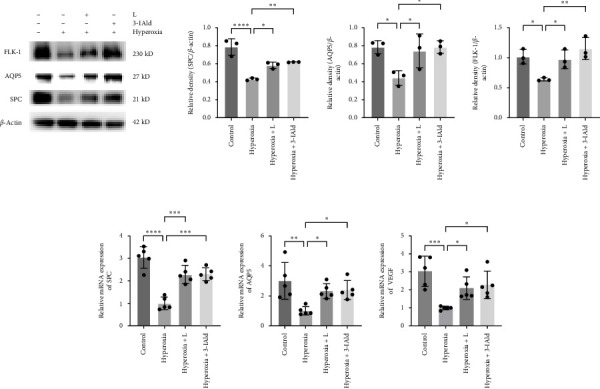
*L. reuteri* increases the expression of SPC, AQP5, FLK-1, and VEGF in mice. (A) Western blot analysis of SPC, AQP5, and FLK-1 expression in the control, hyperoxia, hyperoxia+L, and hyperoxia+3-IAld groups (*n* = 3). (B) qRT-qPCR was used to assess the relative mRNA expression of SPC, AQP5, and VEGF in each group (*n* = 5). The data are presented as means ± SDs. All the statistical analyses were performed by ordinary one-way ANOVA following normality and homogeneity tests. *⁣*^*∗*^*p* < 0.05, *⁣*^*∗∗*^*p* < 0.01,*⁣*^*∗∗∗*^*p* < 0.001, *⁣*^*∗∗∗∗*^*p* ≤ 0.0001. 3-IAld, indole-3-aldehyde; AQP5, aquaporin 5; SPC, surfactant-associated protein C; VEGF, vascular endothelial growth factor.

SPC and AQP5 are markers of alveoli development and FLK-1 is marker of pulmonary vessels development. VEGF is a ligand of FLK-1, that is essential for pulmonary vascular development. In this study, we evaluated the development of alveolar and pulmonary vessels by measuring the expression of SPC, AQP5, FLK-1, and VEGF in mouse lung tissues. Western blotting showed the protein expression of AQP5, SPC, and FLK-1 in the hyperoxia group was significantly lower than those in the control group (Figure 2A). Compared to that in the hyperoxia group, the expression of AQP5, SPC, and FLK-1 was higher in the hyperoxia+L group and hyperoxia+3-IAld group. The results of qPCR showed the same trend, compared with the hyperoxia group, we found the mRNA expression levels of AQP5, SPC, and VEGF in the control group, the hyperoxia+L group and hyperoxia+3-IAld group were significantly increased (Figure 2B).

### 3.3. *L. reuteri* and 3-IAld Upregulate the Expression of IL-22

**Figure 3:**
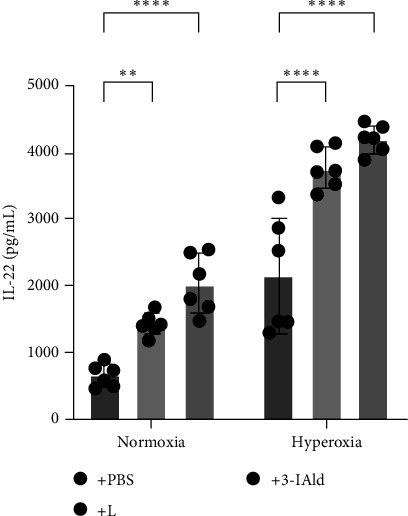
*L. reuteri* and 3-IAld upregulate IL-22 expression in mice. ELISA of IL-22 expression levels in mice treated with PBS, *L. reuteri* or 3-IAld under normoxia (21% O_2_) or hyperoxia (85% O_2_) (*n* = 6). The statistical analyses were performed by ordinary two-way ANOVA. *⁣*^*∗*^*p* < 0.05, *⁣*^*∗∗*^*p* < 0.01,*⁣*^*∗∗∗*^*p* < 0.001, *⁣*^*∗∗∗∗*^*p* ≤ 0.0001. 3-IAld, indole-3-aldehyde; ELISAs, enzyme-linked immunosorbent assay.

In this study, we found significant increased expression of IL-22 in the *L. reuteri*- and 3-IAld-treated groups than in the untreated group (Figure 3). IL-22 significantly improves lung epithelial cell recovery after damage [25], which might explain the protective effects of *L. reuteri* and 3-IAld in hyperoxia-induced mice. Interestingly, hyperoxia exposure alone also stimulated the secretion of IL-22, which may be associated with the activation of the self-repair system after lung damage. The efficiency of the repair system was enhanced by *L. reuteri* and 3-IAld by increasing IL-22 production.

### 3.4. IL-22 Ameliorates BPD-Like Changes in Hyperoxia-Induced Mice

**Figure 4:**
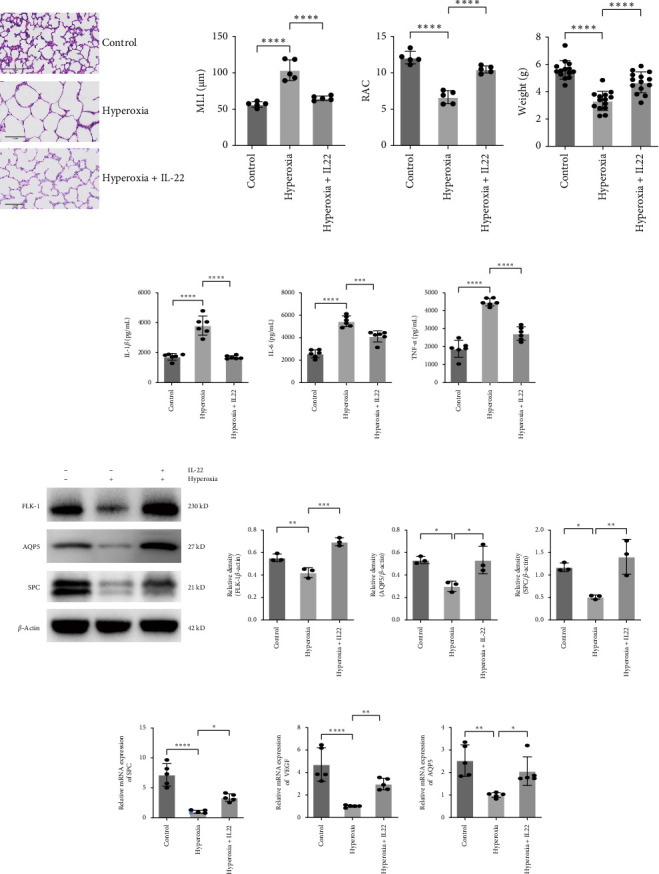
IL-22 has a protective effect against hyperoxia-induced lung injury in mice. (A) H&E staining of lung tissue from the control, hyperoxia, and hyperoxia+IL-22 groups (400×). The MLI and RAC were measured to evaluate alveolarization in each group. The MLI and RAC values are the average of five visual fields in the same section (*n* = 5). (B) Comparison of mouse body weights at P17 showed that IL-22 treatment reduced weight loss (*n* = 14). (C) ELISAs were used to measure the expression levels of IL-1*β*, IL-6, and TNF-*α* in the control, hyperoxia, and hyperoxia+IL-22 groups (*n* = 6). (D) Western blot analysis of SPC, AQP5, and FLK-1 expression in the control, hyperoxia, and hyperoxia+IL-22 groups (*n* = 3). (E) RT-qPCR was used to determine the relative mRNA expression of SPC, AQP5, and VEGF in the control, hyperoxia, and hyperoxia+IL-22 groups (*n* = 5). The data are presented as means ± SDs. All the statistical analyses were performed by ordinary one-way ANOVA following normality and homogeneity tests. *⁣*^*∗*^*p* < 0.05, *⁣*^*∗∗*^*p* < 0.01, *⁣*^*∗∗∗*^*p* < 0.001, *⁣*^*∗∗∗∗*^*p* ≤ 0.0001. AQP5, aquaporin 5; ELISAs, enzyme-linked immunosorbent assays; MLI, mean linear intercept; RAC, radical alveolar count; SPC, surfactant-associated protein C; VEGF, vascular endothelial growth factor.

IL-22 plays a beneficial role in many lung inflammatory diseases [26, 27]. To confirm whether IL-22 plays protective effect against BPD, recombinant IL-22 was intraperitoneally injected into hyperoxia-induced mice. H&E staining showed that IL-22 alleviated alveolar simplification in hyperoxia-induced mice, significantly decreased MLI and increased RAC (Figure 4A). Weight of mice in the hyperoxia+IL-22 group was higher than that in the hyperoxia group (Figure 4B). ELISA results showed the levels of lung proinflammatory cytokinesIL-1*β*, IL-6, and TNF-*α* in the hyperoxia+IL-22 group were significantly lower than those in in the hyperoxia group (Figure 4C). In addition, we found that, compared with the hyperoxia group, the protein expression levels of SPC, AQP5, and FLK-1 and the mRNA expression levels of SPC, AQP5, and VEGF in the hyperoxia+IL-22 group were elevated (Figure 4D,E), suggesting IL-22 contributed to the development of alveolar and pulmonary vessels in hyperoxia-induced mice.

### 3.5. Anti-IL-22 Neutralizing Antibody Inhibits the Protection of *L. reuteri* Against Hyperoxia-Induced Lung Injury

**Figure 5:**
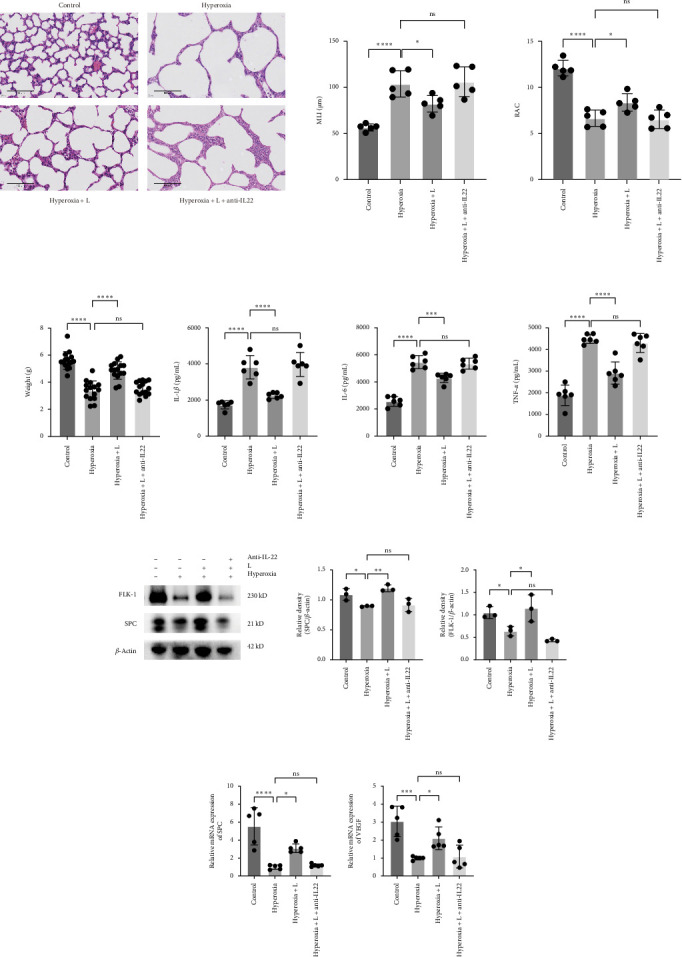
Anti-IL-22 neutralizing antibody inhibits the protective effect of *L. reuteri* on hyperoxia-induced lung injury in mice. (A) H&E staining of lung tissue from the control, hyperoxia, hyperoxia+L, and hyperoxia+L+anti-IL-22 groups (400×). Comparison of the MLI and RAC values for each group. The MLI and RAC values are the average of five visual fields in the same section (*n* = 5). (B) Comparison of the body weights of the mice in each group (*n* = 15). (C) Comparison of the expression levels of IL-1*β*, IL-6, and TNF-*α* in each group (*n* = 6). (D) Western blot analysis of SPC and FLK-1 expression in the control, hyperoxia, hyperoxia+L, and hyperoxia+L+anti-IL-22 groups (*n* = 3). (E) RT-qPCR results showing SPC and VEGF expression in the control, hyperoxia, hyperoxia+L, and hyperoxia+L+anti-IL-22 groups (*n* = 5). The data are presented as means ± SDs. All the statistical analyses were performed by ordinary one-way ANOVA following normality and homogeneity tests. *⁣*^*∗*^*p* < 0.05,*⁣*^*∗∗*^*p* < 0.01, *⁣*^*∗∗∗*^*p* < 0.001, *⁣*^*∗∗∗∗*^*p* ≤ 0.0001. MLI, mean linear intercept; ns, not significant; RAC, radical alveolar count; SPC, surfactant-associated protein C.

The protective effect of *L. reuteri* against hyperoxia-induced lung injury has been demonstrated in the abovementioned experiments. We used an anti-IL-22 neutralizing antibody to determine the role of IL-22 in the protective effect of *L. reuteri*. *L. reuteri* and anti-IL-22 neutralizing antibodies were combined to treat hyperoxia-induced mice, and we found that there was no significant difference in lung pathology (Figure 5A), weight (Figure 5B), and inflammatory cytokine levels (Figure 5C) between mice in the hyperoxia group and those in the hyperoxia+L+anti-IL-22 group. In addition, the results of western blot showed similar expression levels of SPC and FLK-1 in the hyperoxia+L+anti-IL-22 group and hyperoxia group (Figure 5D). Moreover, the qRT-PCR results showed no significant difference in the mRNA expression levels of SPC or VEGF between the two groups (Figure 5E). These results suggested that anti-IL-22 neutralizing antibody inhibited the protective effect of *L. reuteri* in hyperoxia-induced mice.

### 3.6. IL-22 Activates the pSTAT3 Signaling Pathway in MLE-12 Cells

**Figure 6:**
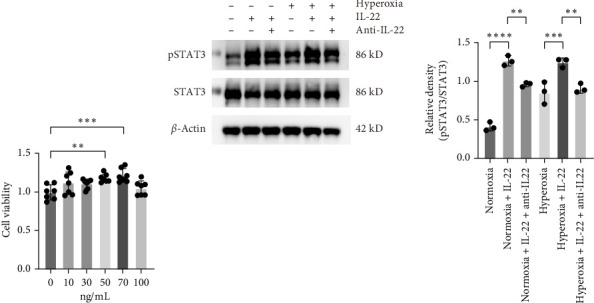
IL-22 activates the pSTAT3 signaling pathway in MLE-12 cells. (A) Cell viability assessment of cells after treatment with different concentrations (0, 10, 30, 50, 70, and 100 ng/mL) of IL-22 (*n* = 7). (B) Western blot analysis of STAT3 and pSTAT3 expression in the normoxia, normoxia+IL-22, normoxia+IL-22+anti-IL-22, hyperoxia, hyperoxia+IL-22, and hyperoxia+IL-22+anti-IL-22 groups (*n* = 3). The data are presented as means ± SDs. All the statistical analyses were performed by ordinary one-way ANOVA following normality and homogeneity tests. *⁣*^*∗*^*p* < 0.05, *⁣*^*∗∗*^*p* < 0.01, *⁣*^*∗∗∗*^*p* < 0.001, *⁣*^*∗∗∗∗*^*p* ≤ 0.0001, ns, not significant.

Cell viability was assessed to determine the optimal concentration of IL-22, and the results showed that the minimal concentration of IL-22 needed to treat hyperoxia-treated MLE-12 cells was 50 ng/mL (Figure 6A). To further explore the potential mechanism, we treated MLE-12 cells under normoxic and hyperoxic conditions with IL-22 and anti-IL-22 neutralizing antibody, and then detected the expression of STAT3 and pSTAT3 by western blot. We found that the ratio of pSTAT3/STAT3 was significantly enhanced in IL-22-treated groups compared to untreated groups (Figure 6B). However, the ratio of pSTAT3/STAT3 was significantly decreases after intervention of anti-IL-22 neutralizing antibody in IL-22-treated groups. The results suggested that IL-22 could drive the phosphorylation of STAT3 in MLE-12 cells.

Therefore, we inferred that *L. reuteri* may ameliorates BPD-like changes in hyperoxia-induced mice by activating the IL-22/STAT3 signaling pathway via IL-22 production.

## 4. Discussion

BPD usually occurs in very and extremely preterm infants who require long-term respiratory support. BPD is currently recognized as a clinical syndrome with complex pathogenesis and variable clinical phenotypes, characterized by different mechanisms involving different regions, such as airway, alveolar, and vascular components, which may have different effects throughout life [28]. BPD is responsible for a large number of mortality and short- and long-term morbidity. Due to the significant delay in the clinical diagnosis of BPD, early identification and intervention of BPD is an urgent problem to be solved.

Probiotic *Lactobacillus* can regulate the immune system to prevent or treat a variety of lung diseases, such as acute respiratory distress syndrome (ARDS), chronic obstructive pulmonary disease (COPD), and asthma [11, 13, 14, 29]. Many *Lactobacillus* species, including *L. reuteri*, *L. rhamnosus GG*, *L. paracasei*, *L. gasseri*, *L. casei*, *L. kunkeei*, *L. plantarum*, and *L. fermentum*, have been reported to play protective roles in respiratory diseases [16, 30–36]. 3-IAld is a tryptophan metabolite of *Lactobacillus*, which can activate AhR. *Lactobacillus* plays a protective role in a variety of chronic inflammatory diseases by producing 3-IAld [37, 38]. In this study, *Lactobacillus reuteri* and 3-IAld were selected to treat BPD mice. Although oral administration of *Lactobacillus* is beneficial to prevent respiratory pathogen infection in mice, but in an asthma-related study, the intranasal administration of *L. paracasei* had a better protective effect than did oral administration [39]. Intranasal administration of *Lactobacillus* induced mucosal immunity and provided protection against lung inflammatory [40, 41]. In this study, intranasal administration was used to intervene neonatal mice.

To verify whether *L. reuteri* can alleviate BPD-like changes in mice, we performed H&E staining of lung tissues in each group. After 14-day hyperoxia induced, lung tissues of hyperoxia group mice showed obvious BPD pathological changes such as simplified alveoli and thickened alveolar septum, suggesting successful establishment of BPD model mice. In addition, compared with hyperoxia group, lung pathological changes of mice in hyperoxia+L group and hyperoxia+3-IAld group obvious alleviated, manifested as significantly reduced MLI value and increased RAC value, suggesting *L. reuteri* and 3-IAld could alleviate the BPD-like changes in the lungs of mice induced by hyperoxia.

Due to immature immunity, premature infants are also prone to pulmonary and systemic infections by invasive medical treatment such as mechanical ventilation and invasive surgery. Persistent pulmonary inflammation exposure is an important factor in the occurrence and development of BPD [42]. *Lactobacillus* can produce a variety of metabolites to exert anti-inflammatory activity. In animal experiment, *Lactobacillus* has been shown to relieve lung inflammation induced by HDM and cigarette smoke in mice [43]. In this study, we found *L. reuteri* and 3-IAld significantly decreased the expression of IL-1*β*, IL-6, and TNF-*α* in lung tissues and relieved lung inflammation in mice.

Due to arrested lung development, BPD is characterized by alveolar hypoplasia and disordered pulmonary vascular development [44]. SPC is secreted by AEC2 and plays an important role in reducing the air–liquid plane tension of alveolar and improving lung antegrade. AQP5 is widely distributed in the apical membrane of AEC1 but not expressed in AEC2. It plays an important role in maintaining respiratory water permeability in physiological condition, and in lung diseases, such as acute lung injury, pulmonary edema, COPD, asthma, and the expression of AQP5 decreases [45]. FLK-1 (VEGFR2), the second type receptor of VEGF, is the main receptor in vascular endothelial cells and plays an important role in angiogenesis and vascular permeability [46]. In this study, the effects of *L. reuteri* and 3-IAld on alveolar development and pulmonary vascular development were evaluated by the detection of SPC, AQP5, VEGF, and FLK-1. We found that the expression of alveolar epithelial markers (SPC, AQPC) and pulmonary vascular endothelial markers (VEGF, FLK-1) in lung tissue of mice in hyperoxia+L group and hyperoxia+3-IAld group was significantly higher than that in hyperoxia group. The results suggested that *L. reuteri* contributed to alveolar development and pulmonary vascular development in BPD mice. In short, our study confirmed *L. reuteri* could alleviate hyperoxia-induced mice BPD-like changes in lung tissues. Intranasal administration of *L. reuteri* is beneficial for hyperoxia-induced lung injury in mice.

IL-22 promotes cell proliferation and survival by upregulating the prosurvival genes Bcl-2, Bcl-XL, and Mcl-1, and the proliferative factors c-Myc, cyclin D1, Rb2, and CDK4 [47]. In this study, we found higher expression levels of IL-22 after treatment with *L. reuteri* and 3-IAld. The result suggests that *Lactobacillus* upregulates IL-22 expression, which is consistent with Abigail's findings [23]. IL-22 expression is mainly regulated by AhR, and a variety of tryptophan metabolites, including 3-IAld, can activate AhR [48, 49]. We, therefore, hypothesized that after *L. reuteri* supplementation, indole tryptophan metabolites were increased and AhR ligands accumulated, causing AhR activation and increased IL-22 production and secretion. Notably, the level of IL-22 in the hyperoxia group was greater than that in the control group indicating that hyperoxia was able to induce IL-22 expression [50]. Considering that IL-22 plays a key role in wound healing and tissue regeneration, increasing IL-22 in BPD can be explained by the self-initiation of lung tissue regeneration after damage [51].

IL-22 is produced primarily by innate lymphocytes and adaptive T cells in response to lung damage caused by infections, allergies, and fibrosis [26, 27]. IL-22 plays an important role in mucosal defenses, resisting invasion of a variety of pathogens, such as *Klebsiella pneumoniae*, *P. aeruginosa*, and influenza virus [52–54]. Inhalation of recombinant IL-22 can effectively reduce ventilator-induced lung injury [55]. A COPD study showed that IL-22 mitigated neutrophilic infiltration, apoptosis, and emphysema and prevented a decrease in lung function [56]. Several studies agree that IL-22 can attenuate allergic airway inflammation in an ovalbumin-induced asthma mouse model [57]. However, little is known about the effect of IL-22 on BPD. To confirm the effect of IL-22 on the development of BPD, recombinant IL-22 was intraperitoneally injected into neonatal mice. The protective effect of IL-22 against hyperoxia-induced lung injury was verified by decreased weight loss, decreased lung inflammatory cytokine levels, and increased expression of SPC, AQP5, and FLK-1. According to our study, IL-22 alleviates hyperoxia-induced mice lung BPD-like changes and plays a protective role in BPD.

Anti-IL-22 neutralizing antibodies have been shown to block the functional activity of IL-22 in several in vitro and in vivo studies [58, 59]. To determine whether the protective effect of *L. reuteri* on BPD is mediated by IL-22, *L. reuteri* and anti-IL-22 antibodies were administered to hyperoxia-induced mice, and the results showed that the protective effect of *L. reuteri* on BPD could be inhibited by an anti-IL-22 antibody. These results indicate *L. reuteri* alleviates lung damage caused by hyperoxia through stimulating IL-22 expression.

IL-22 is a member of the IL-10 cytokine family, its classical signaling occurs through Jak1, tyrosine kinase 2 (Tyk2), and STAT3 [60]. STAT3 is critical for hyperoxia-induced lung injury, and STAT3-null mice have increased susceptibility to hyperoxia lung injury and reduced or absent surfactant proteins and lipids in BALF [61, 62]. IL-22/STAT3 signaling promotes proliferation of oral basal epithelial cells [63]. In addition, Liu et al. [64] found that activation of IL-22/STAT3 signaling could alleviate liver injury, inhibit hepatocyte apoptosis, and decrease inflammatory factors. To further explore the mechanism, MLE-12 cells were induced by 95%O_2_ to establish BPD cell model. In this study, we found IL-22 could enhance STAT3 phosphorylation, while anti-IL-22 neutralizing antibody inhibited IL-22's promotion of STAT3 phosphorylation, indicating that IL-22 activated STAT3 signaling pathway in MLE-12 cells. Therefore, we inferred that *Lactobacillus* may play an important role in the development of BPD by activating the IL-22/STAT3 signaling pathway via IL-22 production.

In addition, there are some limitations in our study. First, the BPD animal model adopted in our study is a single-factor hyperoxia-induced model, which cannot fully simulate the pathogenesis and pathological state of BPD. In order to get closer to the real pathological state, we can combine hyperoxia and LPS to construct the two-strike model of BPD. Second, due to 14-day hyperoxia-induced BPD animal model, our study could only evaluate the short-term efficacy of *Lactobacillus* on lung injury in BPD mice, without long-term efficacy analysis. Therefore, we should extend the modeling time to observe the long-term effects of *Lactobacillus* on BPD. Clinically, maternal microbiome, delivery mode, feeding mode, and neonatal respiratory support all may affect the dynamic balance of lung microbiome in newborn, therefore affect the therapeutic effect of *Lactobacillus*. However, these potential confounders are difficult to reflect in animal models, which may lead to deviations in the conversion of the results of animal experiments to the clinic. In addition, not all treatments in this study were treated in the same way, for example, *Lactobacillus* and 3-IAld were treated with intranasal administration, while IL-22 and anti-IL-22 were treated with intraperitoneal injection, and differences in treatment are potential confounding variables that could affect the results.

In conclusion, our study confirmed *L. reuteri* alleviated hyperoxia-induced lung BPD-like changes in mice by activating the IL-22/STAT3 signaling pathway via IL-22 production. Probiotics *Lactobacillus* is a potential treatment for hyperoxia-induced lung injury in newborns.

## Figures and Tables

**Table 1:** Primers for genes amplified by RT-qPCR.

Gene	Primers sequences
SPC-F	CGTGGTGATTGTAGGGGCTC
SPC-R	GCCGATGGAAAAGGTAGCGA
AQP5-F	CAACACAACACCAGGCAAGG
AQP5-R	GAAAGATCGGGCTGGGTTCA
VEGF-F	GGACTTGTGTTGGGAGGAGG
VEGF-R	CCAGGAATGGGTTTGTCGTG
GAPDH-F	TGTGTCCGTCGTGGATCTGA
GAPDH-R	TTGCTGTTGAAGTCGCAGGAG

Abbreviations: AQP5, aquaporin 5; SPC, surfactant-associated protein C; VEGF, vascular endothelial growth factor.

## Data Availability

The datasets utilized and analyzed in this research can be obtained upon request from the corresponding author.
